# Folie à deux

**DOI:** 10.4103/0019-5545.55942

**Published:** 2005

**Authors:** P.N. Suresh Kumar, N. Subramanyam, Biju Thomas, Abu Abraham, Kishore Kumar

**Affiliations:** * Director, Institute of Mental Health and Neurosciences, Kozhikode 673016, Kerala, India; ** Consultant Psychiatrist, Institute of Mental Health and Neurosciences, Kozhikode 673016, Kerala, India; *** Consultant Psychiatrist, Institute of Mental Health and Neurosciences, Kozhikode 673016, Kerala, India; **** Consultant Psychiatrist, Institute of Mental Health and Neurosciences, Kozhikode 673016, Kerala, India; ***** Medical Officer, Institute of Mental Health and Neurosciences, Kozhikode 673016, Kerala, India

**Keywords:** *Folie à deux*, *folie imposé*, induced delusional disorder, shared psychotic disorder

## Abstract

*Folie à deux* is defined as an identical or similar mental disorder affecting two or more individuals, usually the members of a close family. Two case reports of this condition are presented with a brief review of the literature. Prompt recognition of this condition is an essential step in the management. The majority of patients with *folie à deux* require multiple treatments including separation, antipsychotics, individual and group psychotherapy, and family therapy.

## INTRODUCTION

The term *folie à deux* includes several syndromes in which mental symptoms, particularly paranoid delusions, are transmitted from one person to one or more others with whom the apparent instigator is in some way intimately associated so that he or she and they come to share the same delusional ideas. It was described as *folie communiqué* in 1860 by Baillarger and as *folie à deux* in 1877 by Lasegue and Farlet.^1^ Many synonyms have been used for describing this condition, which mainly reflect the idea of the condition's transmissibility, viz. ‘communicated insanity’, ‘contagious insanity’, ‘infectious insanity’, ‘psychosis of association’ and ‘double insanity’. Although this condition involves two people, it can extend from the original subject to three, four, five persons, viz. *folie a trois, folie a quatre, folie a cinq*, or even a whole family, *folie a famille*.^2^

An exact figure for the incidence and prevalence of *folie à deux* is not available. Cases have been reported from beyond western societies, including Nigeria and India.^3^–^7^ It is certainly more common in isolated communities and families where there is a great urge to defend the *status quo*.

*Folie à deux* is undoubtedly an intriguing condition of great relevance to the understanding of human psychopathology. It is perhaps the most impressive example of a pathological relationship and, therefore, an understanding of its underlying mechanism has theoretical implications for other kinds of disturbed interpersonal relationships.

We discuss the case of 2 patients with *folie imposé*, who presented to our OPD.

## CASE I

Two members of a family—a mother and her son—were brought by the woman's brother to the outpatient department of the Institute of Mental Health and Neurosciences, Kozhikode with similar complaints of irritability, social withdrawal and persecutory delusions. Here is a brief outline of both the individuals.

### Patient A (the Principal)

The mother, a 40-year-old divorcee from a low socioeconomic background, with primary school education presented with continuous illness of 8 years' duration interspersed with exacerbations and partial remissions. She accused her neighbours of plotting to do away with her and her son by poisoning their source of drinking water. She also charged them with having used witchcraft and black magic to kill them. She was often found verbally abusing the neighbours at the top of her voice. She stopped visiting them and actively resisted any social calls by them. She even forced her son to stay indoors and cut off all links with their neighbours. She soon included her own family members in this conspirational network when she saw them with the neighbours on a number of occasions. A mental state examination on the day of admission revealed an unkempt and agitated lady clinging to her son. The rapport was poor, and her responses were brief and evasive. She also had persecutory delusions, an anxious mood and third person auditory hallucinations. She was admitted to the hospital and after a detailed evaluation was diagnosed to have paranoid schizophrenia. Physical examination and preliminary investigations were within normal limits.

### Patient B (the Associate)

The second patient was the only child of the primary case, a 20-year-old unmarried man staying with his mother. He started having behavioural changes 4 years back with similar beliefs of being harmed by his neighbours through witchcraft and black magic. He too was found to be withdrawn, aloof and apprehensive about imminent danger. Two months back, he was bitten by a stray dog for which he vehemently refused treatment and hence the neighbours took him by force for antirabies injection. Following this, both the mother and the son had an aggravation of the illness with restlessness, decreased sleep and incessant speech using foul language directed at their neighbours. It was at this stage that they were brought for admission. A mental status examination revealed an equally unkempt and restless person with persecutory delusions but no perceptual abnormalities. Physical examination and preliminary investigations were within normal limits.

There was no past history of mental illness or substance use in both the mother and the son. Both were admitted in two separate closed wards and isolated from each other. The mother was started on antipsychotic treatment, which was titrated to a maximum of 30 mg of trifluperazine and 200 mg of chlorpromazine per day. After two weeks of treatment, she showed improvement and at the end of one month, reached partial clinical remission.

The son was kept under observation with no drugs for nearly a week to further substantiate whether it was a case of imposed (*folie imposé*) or simultaneous (*folie simultanée*) psychosis. But his deteriorating general condition did not permit us to continue this management and warranted active, aggressive medication. He was also started on trifluperazine, which was titrated up to 40 mg per day and chlorpromazine, which was stabilized at 200 mg per day. His condition improved markedly with drugs and remitted fully in one month.

Before they were both discharged from the hospital, the son agreed to stay in his uncle's house, separate from his mother. They were on regular follow-up for 10 months and maintained good remission.

## CASE II

A 30-year-old divorced mother, belonging to the lower socioeconomic group, presented to the outpatient department of the Institute of Mental Health and Neurosciences, Kozhikode with her 8-year-old son who had been referred by the paediatrics department. They were living together, isolated from the rest of their family, and supported themselves mainly by the mother's begging and occasional manual labour.

The mother harboured strong persecutory delusions against her husband and his relatives. She accused her husband of frequently visiting her son in school, and abusing and torturing him physically. She readily showed the normal skin creases of the inguinal region of her son and said that those were entirely scar marks formed as a result of her husband's physical assaults on the child. She also said that her son's kidney (actually pointing to the testicular region) had been removed by her husband. The child also harboured similar delusions and, in a separate interview, he too narrated the same story as his mother and showed the ‘scar marks’. He said that his father and his men (with whom they did not have any contact for many years) regularly visited him and took him away in a jeep to assault and abuse him.

Both the mother and the child were admitted for a detailed evaluation and management after the initial work-up at the OPD. Unfortunately, fearing further interviews and treatment for mental illness, they absconded from the ward on the second day itself and could not be traced.

## DISCUSSION

The importance of *folie à deux* becomes apparent when it is realized how few people in close association with deluded individuals actually do acquire delusions. A large number of patients with schizophrenia live in intimate association with their relatives, yet no such sharing of delusions occurs. Therefore, when it does happen, there is a need to identify the special factors that operate in each individual's case.

In both the above-described cases, the two individuals were staying together and had minimal contact with relatives or friends. In both instances, the mother was dominant and overprotective about her son. She was clearly the one who initiated their shared delusions, which she progressively imposed on her son who by nature was passive, dependent and submissive. These cases are akin to the classic picture described by Lasegue and Falret^1^ and fall under the subtype of *folie imposé*. They also fulfilled all the criteria for a *folie à deux* described by Dewhurst and Todd: (i) definite evidence that the partners had been in intimate association; (ii) a high degree of commonality in the content of delusion, although the formal psychosis may differ; and (iii) unequivocal evidence that the partners share support, and accept each other's delusions.^8^

A key aspect in the aetiology and psychopathology of *folie à deux* is the nature of relationship between the inducer and the induced. Not only was the son intimately associated with the mother, both in physical proximity and emotional intensity, but the mother had also placed extreme restrictions on his contact with the external world. This had cut off all his connections with reality and catalysed the development of the paranoid delusions. This relationship has also been described and explained in terms of learning theory. The induced thus learns the abnormal behaviour from the more dominant, more driving inducer, and becomes psychotic and behaves psychotically. Whether severing the social contact between the two would have caused a remission is a matter that is still in doubt. Since they were first-degree relatives, a common genetic pool predisposing to a similar illness cannot be ruled out as the patient in the first case failed to show improvement by separation alone. Organic factors play a role when the condition occurs in elderly subjects but psychological and environmental factors are obviously of greater aetiological importance than overt organicity.

Psychodynamically, the submissive, induced person unconsciously acquires the characteristic of the more dominant inducer. The dominant partner provokes the submissive one into accepting his/her delusions rather than risk the deterioration of a close and gratifying relationship. *Folie à deux* thus keeps the pair united, but increases their detachment from the world of reality.^2^

Identification is another psychological mechanism of paramount importance in the production of this condition. Deutsch took the matter still further, stating that ‘close living together from the beginning is an expression of those unconscious forms which later bring both parties to a similar delusional idea; the common delusion appears to be an important part of an attempt to rescue the object through identification with it or its delusional system’.^2^

More recent psychodynamic formulations suggest that the outstanding feature of the recipient consists of ambivalence, and a love-hate relationship. Thus, while the recipient is dependent on the inducer for so much, the recipient, at the same time, despises both the inducer and himself/herself on account of this dependency. Despite this, the recipient needs to maintain the relationship and continue to keep the favour of the partner, and maintain continuing submission even to the point of sharing the delusions. Yet, at times, the recipient realizes and will admit to harbouring aggressive feelings towards the other. The same applies to the dominant partner, who too has a need for passive, submissive companion, if only, as already implied, to keep as long as possible in touch with reality, yet this love easily and quickly turns to hate, especially when it can be seen that the partner threatens to desert him/her. Thus, their relationship contains not only dependence but also ambivalence on both sides.

Environmental factors also play a major aetiological role in causation. The very definition of the condition denotes an intimate relationship over the years and a common sharing of delusions when these have become established. Those who live in increasing isolation tend to become increasingly mistrustful, feeling themselves threatened by a seemingly increasingly hostile atmosphere which, in turn, leads to the production of paranoid reaction and eventually paranoid psychosis. *Folie à deux* becomes, therefore, a way of coping with such hostility and aggression. Studies done in the West have noted that social isolation, especially in women, is conducive to the production of psychosis of a paranoid kind.^9^ Others have emphasized the role of imitation and sympathy in the causation of *folie à deux.*^10^ Dewhurst and Todd suggested that in the psychosis of association, the dominant partner uses the powers of suggestion, which in its essentials resembles hypnosis, to convey his/her delusions to the weaker partner, although emphasizing that the essential dominance which is a prerequisite for this to occur arises from several and varied factors, such as superiority in age, intelligence, education and aggressive drive.^8^

*Folie à deux* is not only a colourful and intriguing condition, it also serves to emphasize how rarely psychotic symptoms are actually contagious—almost never, in fact, except under those specific conditions that have been enumerated, especially social isolation. As in all rare cases, the first step to proper management and treatment is proper recognition of the condition and situation. When assessing the condition and situation, it is always important to keep in mind the underlying clinical and social factors which, as described, are important in the production of an environment conducive to the sharing of the delusion.
